# Associations of Chinese diagnosis-related group systems with inpatient expenditures for older people with hip fracture

**DOI:** 10.1186/s12877-022-02865-3

**Published:** 2022-03-01

**Authors:** Zhaolin Meng, Kun Zou, Suhang Song, Huazhang Wu, Youli Han

**Affiliations:** 1 School of Nursing, Capital Medical University, Beijing, China; 2 Department of Pharmacy, West China Second University Hospital, Sichuan University, Chengdu, Sichuan China; 3 Evidence-Based Pharmacy Center, West China Second University Hospital, Sichuan University, Chengdu, Sichuan China; 4 The Taub Institute for Research in Alzheimer’s Disease and the Aging Brain, Columbia University, New York, NY USA; 5 Department of Health Service Management, China Medical University, Shenyang, Liaoning China; 6 Department of Health Management and Policy, School of Public Health, Capital Medical University, NO 10, Xi Toutiao Rd Youanmenwai District, Beijing, 100069 China

**Keywords:** Chinese diagnosis-related group (C-DRG), Hip fracture, Older people, Out-of-pocket (OOP) payment, Inpatient expenditure

## Abstract

**Background:**

Hip fracture is frequent in older people and represents a major public health issue worldwide. The increasing incidence of hip fracture and the associated hospitalization costs place a significant economic burden on older patients and their families. On January 1, 2018, the Chinese diagnosis-related group (C-DRG) payment system, which aims to reduce financial barriers, was implemented in Sanming City, southern China. This study aimed to evaluate the associations of C-DRG system with inpatient expenditures for older people with hip fracture.

**Methods:**

An uncontrolled before-and-after study employed data of all the patients with hip fracture aged 60 years or older from all the public hospitals enrolled in the Sanming Basic Health Insurance Scheme from January 1, 2016 to December 31, 2018. The ‘pre C-DRG sample’ included patients from January 1, 2016 to December 31, 2017. The ‘post C-DRG sample’ included patients from January 1, 2018 to December 31, 2018. A propensity score matching analysis was used to adjust the difference in baseline characteristic parameters between the pre and post samples. Data were analyzed using generalized linear models adjusted for the demographic, clinical, and institutional factors. Robust tests were performed by accounting for time trend, the fixed effects of the year and hospitals, and clustering effect within hospitals.

**Results:**

After propensity score matching, we obtained two homogeneous groups of 1123 patients each, and the characteristic variables of the two matched groups were similar. We found that C-DRG reform was associated with a 19.51% decrease in out-of-pocket (OOP) payments (*p* < 0.001) and a 99.93% decrease in OOP payments as a share of total inpatient expenditure (*p* < 0.001); whereas total inpatient expenditure was not significantly associated with the C-DRG reform. All the sensitivity analyses did not change the results significantly.

**Conclusion:**

The implementation of C-DRG payment system reduced both the absolute amount of OOP payments and OOP payments as a share of total inpatient expenditure for older patients with hip fracture, without affecting total inpatient expenditure. These results may provide significant insights for policymakers in reducing the financial burden on older patients with hip fracture in other countries.

**Supplementary Information:**

The online version contains supplementary material available at 10.1186/s12877-022-02865-3.

## Backgrounds

Hip fracture is the most common orthopedic injury in older people and is associated with considerable financial costs to the healthcare system, which becomes a global significant public health concern [1, 2]. Meanwhile, the incidence of hip fractures increases rapidly in low- and middle-income countries (LMICs) [3]. China, as the largest low- and middle-income country with a rapidly aging global population, has been facing great challenges from an increasing number of hip fractures in older individuals [4, 5]. By 2050, the total number of hip fractures in older people is predicted to be 1.3 million in China [6]. For treatment of a hip fracture, elderly patients in China might have expenses that are several times higher than their annual disposable income per capita, which could exert huge financial burdens on patients and their families [7]. Wang and colleagues found that the average cost of all patients with hip fracture was ¥53,440, which was more than the GDP per capita in 2014 (¥46,629) and 2.65 times the average annual disposable income of 2014 (¥20,167), and patients older than 60 years spent significantly higher hospitalization costs, compared with patients from other age groups [8].

Previous studies showed that overuse of medical services, such as preoperative tests in hip fracture patients were associated with unproportionally high costs [9, 10]. It has been claimed that overuse of medicines and medical services is a serious problem across the world including China, because of distorted provider incentives in the fee-for-service (FFS) system [11, 12]. In the past two decades some countries have introduced prospective payment systems based on diagnosis-related groups (DRG) through incentivizing hospitals to reduce unnecessary services and control hospital expenditures [13]. China also has experimented several DRG payment pilot reforms in recent years [14–16]. However, these DRG pilot reforms were rarely systemic, and yielded mixed results [14–16]. In these DRG payment pilot reforms, healthcare providers may have an incentive to increase the out-of-pocket (OOP) payments to compensate for the losses caused by control over the insurance fund, because the OOP payments for inpatient care were still paid on an FFS basis [17, 18].

In an attempt to reduce OOP payments, China developed a payment system namely “the Chinese diagnosis-related group (C-DRG)” in Sanming. Sanming is a city in southern China, which was famous for embarking on a systemic hospital reform by adjusting payment method, pharmaceutical system, and medical services price in 2012 [19, 20]. The systemic hospital reform in Sanming was appraised by Chinese government and international specialized agencies, such as the World Health Organization, and World Bank [21]. On January 1, 2018, Sanming implemented C-DRG payment system reform simultaneously at all of the 22 public hospitals in the city. In this system, both the insurance reimbursements and patient OOP payments are prospectively determined by DRG. For example, patient OOP payments account for 30% and insurance reimbursements account for 70% of a "bundled" payment for each DRG in secondary hospitals (as for other details of the C-DRG reform, see Additional file 1: Table S1).

In theory, the transparent C-DRG payment system, i.e., including the OOP payments in a predetermined fixed rate of "bundled" payments, would limit shifting costs to patients. Our previous study evaluated the economic effects of C-DRG for critically ill patients requiring ICU admission, and found that C-DRG policy reduced financial burden for critically ill patients [22]. However, to the best of our knowledge, the associations of C-DRG reform with inpatient expenditures for older patients with hip fracture has not previously been studied. Based upon the fact of that huge financial burdens on older adults following hip fracture, evaluating the effectiveness of C-DRG on inpatient expenditures for older patients with hip fracture will provide evidence that strategies to reduce financial barriers, which may be an effective strategy to improve population health. Therefore, the aim of this study was to evaluate the impact of C-DRG on hospital expenditures and OOP payments of older people with hip fracture.

## Methods

### Study design

A quasi-experimental ‘uncontrolled before-and-after’ design was selected to explore the impact of C-DRG on total inpatient expenditure and OOP payments of older people with hip fracture. An uncontrolled before-and-after design enables assessment of the relationship between 2 events or interventions, when a typical control group is not available but a pre-post comparison is possible [23]. This design enabled comparison of changes in total inpatient expenditure and OOP payments for older people with hip fracture before and after C-DRG reform. This study was approved by the medical ethics review board of Capital Medical University. All methods were performed in accordance with the relevant guidelines and regulations.

### Study population and data source

Patients with hip fracture aged 60 years or older admitted to all public hospitals from January 1, 2016 to December 31, 2017 constituted the ‘pre C-DRG’ sample and those from January 1, 2018 to December 31, 2018 were the ‘post C-DRG’ sample. Hip fractures were identified using the primary diagnosis with the code-class S72 of the International Classification of Diseases, 10th Revision (ICD-10). The study population included 3274 patients with hip fracture, which were stratified into two groups according to the year of their index dates (*N* = 2137 before the C-DRG reform from January 1, 2016 to December 31, 2017; and *N* = 1137 after the C-DRG reform from January 1, 2018 to December 31, 2018).

Discharge data on beneficiaries were drawn from the Sanming Basic Health Insurance Scheme Database to identify all patients aged 60 years or older admitted for hip fracture at all 22 public hospitals of Sanming City between January 1, 2016 to December 31, 2018. In recent years, the vast majority of China’s population has been covered by the Basic Health Insurance Scheme, such that the discharge data from the Basic Health Insurance Scheme covers more than 95% of Sanming’s population [24, 25]. The date of admission with diagnosis of hip fracture was defined as the index date. The data for each admission obtained from the database contained information of demographic characteristics, clinical data, and hospital expenses.

### Study outcomes and covariates

Study outcomes were total inpatient expenditure per admission, OOP payments per admission, OOP payments as a share of total inpatient expenditure, and length of stay (LOS). All expenditure variables in our study were adjusted to 2016 Chinese yuan (¥) using the consumer price index [26]. To adjust for differential risk among patients, the following covariates were considered:

1. demographic data, including age, gender, and insurance types (Urban and Rural Resident Basic Medical Insurance [URRBMI] and Urban Employee Basic Medical Insurance [UEBMI]); URRBMI covers the urban non-employed and self-employed population and rural population, while the UEBMI covers the urban employed population and retired people;
2. clinical data, including fracture location (femoral neck; trochanteric region of the femur, including trochanteric and subtrochanteric), treatment methods (hip replacement/internal fixation; other treatments including external fixation, bone traction, and other conservative treatments), and the severity of comorbidities estimated using the Charlson comorbidity index (CCI) and dichotomized to less than 3 and equal to or greater than 3 [27];
3. institutional factors, including hospital levels (tertiary hospital and secondary hospital) and hospital types (traditional Chinese medicine [TCM] hospitals and general hospitals).

### Data analysis

The data were described by period (pre C-DRG, post C-DRG), using means for continuous variables and frequencies for categorical variables. And independent samples *t* tests were employed for continuous variables and Chi-square tests for categorical variables between the preintervention and postintervention groups. To balance the two groups in key covariates before and after the implementation of the C-DRG, propensity score matching was performed using maximal exact matches. After the matching, generalized linear models (GLM) were used to evaluate changes of the outcomes before-after the C-DRG reform, controlling for the covariates as described above. Specifically, we used the generalized linear regressions fitted by the least-square approach with the following empirical specification:

$$Y_i=\beta_0+\beta_1C-DRG_i+\beta_2Age_i+\beta_3Gender_i+\beta_4Insurance_i+\beta_5Fracture_i+\beta_6Treatment_i+\beta_7CCI_i+\beta_8Hospital.level_i+\beta_9Hospital.type_i+\varepsilon_i$$

where *Y*_*i*_ represents the outcome variables: total inpatient expenditure per admission, OOP payments per admission, OOP payments as a share of total inpatient expenditure, and LOS. Total spending, OOP payments, and LOS were not normally distributed, and were log-transformed for analysis in the GLM. The coefficient $\beta 1$ estimates the effects of the C-DRG reform on the outcome variables; which represents the difference between ‘before’ and ‘after’ the C-DRG reform was implemented. The coefficients from $\beta 2$ to $\beta 9$ represent a series of covariates, including age, gender, insurance types, fracture location, treatment methods, CCI, hospital levels, and hospital types; *ε*_*i*_ refers to the error term.

The outcome variables were log-transformed in GLM, and the adjusted percentage changes in the outcome variables were calculated by exponentiating the respective regression parameter estimates (*β*_*i*_) according to the following formula [28]:

$$Adjusted\;percentage\;changes\:=\:100\:\times\:(exp\lbrack\beta i\rbrack-1)$$

Three sensitivity analyses were performed to assess the robustness of the findings. First, the GLM analyses were conducted excluding the secular time trend and seasonal factors, and treating the data over the 36-month study time frame as a short time series [29]. Second, the fixed effects of the year and hospitals were controlled to adjust for unobserved underlying factors that change over time and unobserved hospital-specific effects. Third, standard errors were adjusted for clustering of patients within hospitals to account for interactions among groups of patients in the same hospital [30].

Data analyses were performed using SAS (SAS Institute Inc., Cary, NC, USA; version 9.3) and SPSS (IBM Corp., Armonk, NY, USA; version 25). All tests were two-sided, and the significance level was set at 0.05.

## Results

### Descriptive analysis

The demographics and disease characteristics of the patients before and after propensity score matching are summarized in Table 1. The two groups of patients were similar in raw data of age, CCI, proportion in secondary hospitals, and percentage in general hospitals. The two groups varied from the raw data of gender, insurance types, fracture location, and treatment methods. For example, after the C-DRG reform, the percentage of patients treated by surgical treatments (hip replacement or internal fixation) increased from 73.6% to 81.4%, and the proportion of patients with the UEBMI increased from 11.8% to 19.4%. Propensity score matching was performed to adjust for differences in the characteristic variables, and after the matching, we obtained two balanced groups of 1123 patients each. All the included covariates achieved balance after matching.

**Table 1:** Characteristics of the study population before and after propensity score matching

Variables	Before matching			After matching		
	Before C-DRG (*n* = 2137)	After C-DRG (*n* = 1137)	*p*^a^	Before C-DRG (*n* = 1123)	After C-DRG (*n* = 1123)	*p*^a^
Age years, mean (SD)	78.93(8.12)	79.33(8.06)	0.204	79.02(7.95)	79.33(8.06)	0.408
^b^Gender, n (%)			0.007			1.000
Male	799 (37.4)	371 (32.6)		357(31.8)	357(31.8)	
Female	1338 (62.6)	766 (67.4)		766(68.2)	766(68.2)	
^b^Insurance type, n (%)			< 0.001			1.000
URRBMI	1884 (88.2)	916 (80.6)		916(81.6)	916(81.6)	
UEBMI	253 (11.8)	221 (19.4)		207(18.4)	207(18.4)	
^b^Fracture location, n (%)			< 0.001			1.000
Femoral neck	1182 (55.3)	497 (43.7)		497(44.3)	497(44.3)	
Trochanteric region	955 (44.7)	640 (56.3)		626(55.7)	626(55.7)	
^b^Treatment methods, n (%)			< 0.001			1.000
HR/IF	1572(73.6)	925 (81.4)		911(81.1)	911(81.1)	
Other treatments	565 (26.4)	212 (18.6)		212(18.9)	212(18.9)	
Charlson Comorbidity Index,n(%)			0.412			0.951
< 3	1860 (87.0)	978 (86.0)		968(86.2)	967(86.1)	
≥ 3	277 (13.0)	159 (14.0)		155(13.8)	156(13.9)	
Hospital level, n (%)			0.920			0.307
Tertiary hospital	524 (24.5)	277 (24.4)		293(26.1)	272(24.2)	
Secondary hospital	1613(75.5)	860 (75.6)		830(73.9)	851(75.8)	
Hospital type, n (%)			0.406			0.675
General hospital	1682(78.7)	909 (79.9)		890(79.3)	898(80.0)	
TCM hospital	455 (21.3)	228 (20.1)		233(20.7)	225(20.0)	

*Abbreviations*: *SD* Standard deviation, *C-DRG* Chinese diagnosis-related group, *UEBMI* Urban Employee Basic Medical Insurance, *URRBMI* Urban and Rural Resident Basic Medical Insurance, *HR/IF* Hip replacement/internal fixation, *TCM* Traditional Chinese medicine

^a^Results of independent samples *t* test for continuous variables and Chi-square tests for categorical variables

^b^These variables were included in the propensity score matching estimators, by giving priority to exact matches

### Effect of C-DRG on inpatient expenditures

As shown in Table 2, the unadjusted OOP payments per admission (¥9587 vs. ¥7618, *p* < 0.001) and OOP payments as a share of total inpatient expenditure (42.17% vs. 34.44%, *p* < 0.001) significantly decreased after the C-DRG reform, while no significant change was observed in total inpatient expenditure per admission (*p* = 0.059). LOS had a slight decrease after the C-DRG reform (18.89 days vs. 17.48 days, *p* = 0.002). Regarding the inpatient expenditures by components of services, drug expenditure (¥2077 vs. ¥1718, *p* = 0.033) and physician services and therapeutic services expenditure (¥19,550 vs. ¥16,822, *p* = 0.008) decreased significantly; whereas no significant change occurred in diagnostic testing expenditure after the C-DRG reform (*p* = 0.830). (In 2016 a US dollar was equivalent to 6.6 Chinese yuan).

**Table 2:** Unadjusted inpatient expenditures and length of stay before and after C-DRG reform

	Before C-DRG Mean (SD)	After C-DRG Mean (SD)	*p*^a^
Total inpatient expenditure^b^ (¥)	22,287 (13,595)	21,252 (12,400)	0.059
OOP payments^b^ (¥)	9587 (6643)	7618 (5724)	< 0.001
OOP payments as a share of total expenditure (%)	42.17 (10.06)	34.44 (9.15)	< 0.001
Length of stay (days)	18.89 (12.19)	17.48 (9.60)	0.002
Drug expenditure^b^ (¥)	2077 (1608)	1718 (1749)	0.033
Diagnostic testing expenditure^b^ (¥)	2742 (1605)	2712 (1421)	0.830
Physician services and therapeutic services expenditure^b^ (¥)	19,550 (10,093)	16,822 (10,586)	0.008

*Abbreviations*: *C-DRG* Chinese diagnosis-related group, *SD* Standard deviation, *OOP* Out-of-pocket

^a^Results of independent samples *t* test

^b^Exchange rate: 6.6 Chinese yuan (¥) to US $ 1.0

As listed in Table 3, after adjusting for the covariates in the GLM analyses, the OOP payments per admission had a 19.51% (= exp^−0.217^–1, *p* < 0.001) decrease, and OOP payments as a share of total inpatient expenditure decreased by 99.93% (= exp^−7.335^–1, *p* < 0.001) after the C-DRG reform. Moreover, there was a slight decrease in LOS by 8.33% (= exp^−0.087^–1, *p* < 0.001), while no significant change was observed in total inpatient expenditure per admission.

**Table 3:** The GLM analysis results of inpatient expenditures and length of stay

Variable	Ln (Total expenditure)	Ln (OOP payments)	Ln (OOP% of total expenditure)	Ln (Length of stay)
C-DRG reform (after vs. before^ref^)	− 0.035 (0.022)	− 0.217 (0.026)^***^	− 7.335 (0.373)^***^	− 0.087 (0.022)^***^
Age	0.003 (0.001)	− 0.0004 (0.002)	− 0.087 (0.024)^***^	− 0.003 (0.001)
Gender (female vs. male^ref^)	0.003 (0.025)	− 0.019 (0.028)	− 0.279 (0.409)	0.001 (0.024)
Insurance types (URRBMI vs. UEBMI^ref)^	− 0.048 (0.029)	0.212 (0.033)^***^	9.151 (0.482)^***^	− 0.142 (0.029)^***^
Fracture location (femoral neck vs. trochanteric^ref^)	0.216 (0.023)^***^	0.242 (0.027)^***^	1.053 (0.388)^**^	0.070 (0.023)^**^
Treatment (HR/IF vs. others^ref)^	1.899 (0.029)^***^	1.936 (0.033)^***^	0.977 (0.485)^*^	1.098 (0.029)^***^
Charlson Comorbidity Index (≥ 3 vs. < 3^ref^)	− 0.014 (0.031)	− 0.045 (0.035)	− 0.736 (0.510)	0.014 (0.030)
Hospital level (tertiary vs. secondary^ref^)	0.181 (0.027)^***^	0.462(0.030)^***^	10.909 (0.446)^***^	0.035 (0.027)
Hospital type (TCM vs. general^ref^)	− 0.006 (0.027)	0.013 (0.031)	0.619 (0.456)	− 0.012 (0.027)
Intercept	7.874 (0.127)^***^	6.881 (0.145)^***^	37.008 (2.121)^***^	2.167 (0.126)^***^
R-square	0.706	0.679	0.377	0.461

*Abbreviations*: *C-DRG* Chinese diagnosis-related group, *GLM* Generalized linear models, *ref* Reference group, *OOP* Out-of-pocket, *UEBMI* Urban Employee Basic Medical Insurance, *URRBMI* Urban and Rural Resident Basic Medical Insurance, *HR/IF* Hip replacement/internal fixation, *TCM* traditional Chinese medicine

Standard errors in parentheses. ^*^p < 0.05, ^**^p < 0.01, ^***^p < 0.001

### Sensitivity analysis

First, sensitivity analyses showed that the results were similar to the main analysis when excluding the secular time trend and seasonal factors (Table 4). Further, as shown in columns (1)–(4) of Additional file 2: Table S2, controlling for the fixed effects of the year and hospitals yielded similar results: OOP payments, OOP payments as a share of total inpatient expenditure, and LOS decreased significantly by 15.97% (= exp^−0.174^–1, *p* < 0.001), 99.85% (= exp^−6.471^–1, *p* < 0.001), and 10.42% (= exp^−0.110^–1, *p* < 0.001), respectively, while no significant change was observed in total inpatient expenditure. In addition, after inclusion of the cluster standard errors by hospital, the estimated coefficients reported in columns (5)–(8) of Additional file 2: Table S2 were also similar to that in the main analysis.

**Table 4:** The GLM analysis results of inpatient expenditures and length of stay: Adjusting for the secular trend

Variable	Ln (Total expenditure)	Ln (OOP payments)	Ln (OOP% of total expenditure)	Ln (Length of stay)
C-DRG reform (after vs. before^ref^)	− 0.008 (0.041)	− 0.093 (0.046)^*^	− 3.748 (0.672)^***^	− 0.223 (0.040)^***^
Age	0.003 (0.001)	− 0.0007 (0.002)	− 0.095 (0.024)^***^	− 0.002 (0.001)
Gender (female vs. male^ref^)	0.003 (0.025)	− 0.020 (0.028)	− 0.338 (0.406)	0.005 (0.024)
Insurance types (URRBMI vs. UEBMI^ref)^	− 0.052 (0.029)	0.196 (0.033)^***^	8.695 (0.483)^***^	− 0.126 (0.029)^***^
Fracture location (femoral neck vs. trochanteric^ref^)	0.215 (0.023)^***^	0.233 (0.027)^***^	0.793 (0.386) ^*^	0.080 (0.023)^***^
Treatment (HR/IF vs. others^ref)^	1.897 (0.029)^***^	1.931 (0.033)^***^	0.831 (0.480)	1.103 (0.029)^***^
Charlson Comorbidity Index (≥ 3 vs. < 3^ref^)	− 0.011 (0.031)	− 0.035 (0.035)	− 0.437 (0.508)	0.002 (0.030)
Hospital level (tertiary vs. secondary^ref^)	0.180 (0.027)^***^	0.454 (0.031)^***^	10.675 (0.444)^***^	0.044(0.027)
Hospital type (TCM vs. general^ref^)	− 0.007 (0.027)	0.013 (0.031)	0.644 (0.452)	− 0.012 (0.027)
Intercept	7.942 (0.155)^***^	7.150 (0.175)^***^	44.103 (2.547)^***^	1.937 (0.152)^***^
Time trend	− 0.002 (0.002)	− 0.008 (0.003)^**^	− 0.237 (0.037)^***^	0.009 (0.002)^***^
September^a^	− 0.027 (0.042)	− 0.078 (0.048)	− 1.733 (0.700)^*^	0.054 (0.042)
October^a^	− 0.004 (0.043)	− 0.027 (0.048)	− 0.653 (0.703)	− 0.002 (0.042)
R-square	0.706	0.681	0.391	0.466

*Abbreviations*: *C-DRG* Chinese diagnosis-related group, *GLM* Generalized linear models, *ref* reference group, *OOP* Out-of-pocket, *UEBMI* Urban Employee Basic Medical Insurance, *URRBMI* Urban and Rural Resident Basic Medical Insurance, *HR/IF* Hip replacement/internal fixation, *TCM* Traditional Chinese medicine

Standard errors in parentheses. ^*^p < 0.05, ^**^p < 0.01, ^***^p < 0.001

^a^September, October: indicators for inpatient admissions in these months

## Discussion

To the best of our knowledge, this is the first study to evaluate the associations of C-DRG with inpatient expenditures for elderly people with hip fracture, using real-world insurance individual level data. This study showed that C-DRG reform was associated with a decrease in both the absolute amount of OOP payments and OOP payments as a share of total inpatient expenditure. There was also a slight decrease in LOS, though no significant difference was observed for total inpatient expenditure. Results from sensitivity analysis also supported the findings in main analysis.

This study showed that in older patients with hip fracture, C-DRG payment reform was associated with a 19.51% decrease in OOP payments per admission and a 99.93% decrease in OOP payments as a share of total inpatient expenditure. This would significantly relieve financial burdens for older patients with hip fracture and their families. These findings were expected in accordance with the economic theory of provider behavior [31]. Under the previous payment system in Sanming, some services items for hip fractures, such as anesthesia analgesic pump and absorbable suture were not covered by the Basic Health Insurance Scheme, and therefore, patients had to pay 100%. And thus, it was reasonable to assume that healthcare staff might focused on OOP payments by providing those services items outside of the reimbursement list to generate revenue from OOP payments [31]. The previous reimbursement system offered the providers a 'perverse incentive' for cost shifting to the patient to protect the hospital income. A 'perverse incentive' is an incentive that motivates behavior just like other incentives, but also results in unintended negative consequences, which is often seen with financial incentives in healthcare [32]. A previous study in Korea also suggested that healthcare staffs had financial incentives to substitute health services that were not reimbursed by a stringent payment, with the potential of increasing OOP cost burden for patients [18]. Consistent with theoretical predictions, under the C-DRG system, transparent OOP payments, which were based on a predetermined fixed rate of "bundled" payment for each admission, reduced the potential for cost-shifting to patients and protected patients from unpredicted hospital expenditures.

Previous studies showed that in many LMICs, such as Malaysia, Vietnam, and Nigeria, excessive reliance on OOP payments led to financial barriers or impoverishment, especially among the most vulnerable [33–37]. It was reported that half of the world’s population could not still obtain essential health services and over 100 million were pushed into extreme poverty by paying OOP payments for care [38]. Better financial risk protection is one of the universal health coverage (UHC) goals and remains a challenge for most LMICs. Research on how to control the OOP payments is extremely vital to achieve the UHC goal in LMICs. The C-DRG reform in China may provide potentially valuable evidence to other LMICs to provide better financial protection for the vulnerable people, such as older patients with hip fracture.

The patients were divided into two groups according to their treatment methods in this study, the surgically treated types (internal fixation or hip replacement) and other treatments. The later included mostly patients who received the swelling and pain medications, and a variety of nonoperative treatments, such as bone traction and external fixation. This group accounted for 26.4% before the C-DRG reform, and 18.6% after the C-DRG reform. It is noteworthy that the utilization of inpatient surgical treatment (hip replacement or internal fixation) for older patients with hip fracture increased after the C-DRG reform. A potential explanation might be that a dramatic decrease in OOP payments could lead to better financial access to inpatient surgery, and contribute to some increase in the need of some discretionary surgeries among vulnerable populations. A previous study [39] showed that insurance expansion led to greater utilization of elective inpatient procedures that were often performed to improve quality of life. And thus, we assumed that the increase in surgical treatment in our study further implied that C-DRG reduced the financial burden for older patients with hip fracture. But it was not clear whether the increase in utilization of such surgeries represented a response to unmet need or changes in treatment thresholds driven by patients or physicians, which should be examined in future studies. Moreover, it is important to note that we couldn’t have a direct causal interpretation that the increase in surgical treatment might be the result of the change in financing without a control group. There were other factors which might influence this change. For example, the proportion of patients who had UEBMI increased from 11.8% to 19.4% after the C-DRG reform (Table 1). And in this study, it was shown that patients covered by UEBMI had a higher proportion of surgery (75.3% vs.81.9%, see Additional file 3: Table S3) compared with URRBMI. This variation in utilization of surgery between patients covered by URRBMI and by UEBMI might be due to benefit differences of the two insurance types. There were more benefits, such as a higher reimbursement rate for inpatient care under the UEBMI compared to that under the URRBMI. Meanwhile, the urban employed population who covered by UEBMI usually have a higher income than the urban non-employed and rural residents covered by URRBMI [24].

It is also noteworthy that although the financial burden on older patients with hip fracture declined significantly after the C-DRG reform, OOP payments as a share of total inpatient expenditure after the reform was still 34.38%, which is higher than that in high-income countries (generally below 25%) [40]. According to the 2018 World Health Organization (WHO) report, OOP payments between 2000 and 2015 decreased from 23% of current health expenditure to 21% in high-income countries [40]. Therefore, China still has room to improve in terms of the benefits that older patients with hip fracture may enjoy from the social health insurances.

In terms of LOS, there was a slight decrease after the C-DRG reform, but the estimated effect was small: the unadjusted LOS only decreased around a day after the C-DRG reform (18.89 days before C-DRG vs. 17.48 days after C-DRG), and an 8.42% decrease for the LOS in the GLM analysis also means about 1 day for a 15-day stay. This result implied that a slightly shorter LOS might not be a main contributor to the savings of OOP payments. The dramatic decrease in OOP payments was largely accomplished through the C-DRG reform strategies (for the details of the C-DRG reform measures, see Additional file 1: Table S1). The LOS of the group of surgically treated patients (20.42 ± 10.00) was higher than that of other treatments (8.86 ± 11.03) (see Additional file 4: Table S4), which might be due to the fact that surgically treated patients usually need longer time of exercise rehabilitation services in the hospitalization, without going to rehabilitation facilities in China. The patients with hip fracture were usually discharged to home, instead to rehabilitation facilities, which might be due to strong family support in China for elderly patients. And, in our study, conservative treatment included mostly patients who did not receive treatment other than the swelling and pain medications. The low proportion of patients treated by traction might be an important reason for the shorter LOS among patients with other treatments, compared to the LOS among the surgically treated patients.

For the inpatient spending by type of services, drug expenditures and the expenditures for physician services and therapeutic services decreased significantly after the C-DRG reform, implying that C-DRG reform changed health care providers’ behavior. In our previous study, orthopedic surgeons in Sanming indicated that they were more enthusiastic to consider cost-effectiveness while determining available treatments for patients with hip fracture after the C-DRG reform [41]. A previous survey also suggested that most physicians agreed to take a more prominent role in limiting the use of unnecessary tests and expensive treatments with little net benefit [42]. These results implied that the C-DRG reform resulted in reduced intensity of inpatient care in terms of medication usage, supply of physician services, and therapeutic services; however, it cannot be stated with certainty whether the reduction in the intensity of care mainly resulted from reducing low-value or high-value care services. Low-value care is care for which there is evidence that it has no or little benefit to the patient, causes harm and wastes limited resources [43]. In recent years, some researchers developed algorithms measuring low-value care services using claims data [43]. Future studies on the association between the prospective-based payment of C-DRG and low-value care services may help to extend our understanding for this mechanism.

In this study, the adoption of C-DRG was associated with a significant decline in OOP payments, but not total inpatient expenditure. This result implied that older patients with hip fracture were provided with better financial protection through more extensive insurance reimbursements. The lack of change in total inpatient expenditure after the C-DRG reform could be explained by specific measures designed to prevent healthcare providers from lowering the standard of treatment under the C-DRG system, which warranted the quality of treatments. The main measures to ensure a high quality of care included: 1) encouraging the use of clinical pathways and guidelines; 2) using a robust health information system to detect readmission rates and discharge; 3) rating the performance of directors at each hospital based on a set of indicators that included clinical quality, and tying the incomes of healthcare staff to the performance of the directors. These measures might motivate healthcare providers to proactively keep and improve the quality of care to obtain full payment. Under the incentive mechanisms, cost-saving incentives for healthcare providers might be weakened; nevertheless, the risk of undertreatment due to healthcare providers’ intention to contain costs was reduced. Some researchers concerned that DRG payment systems may compromise quality of care as the prospective payment system of transfer price controls on hospital payments led to cost-cutting measures by hospitals [44, 45]. Measuring the quality of care has often been a challenge. Hospital mortality rates or readmission rates are frequently cited as measures of quality of care. Several studies have examined whether the adoption of DRG payment system in China impacted the quality of care in terms of in-hospital mortality or readmission rates, and they showed that DRG payment systems did not affect outcomes of in-hospital deaths or readmission rates [14, 44]. In this study, as shown in Additional file 5: Table S5, the overall quality indicators of public hospitals in Sanming, including inpatient mortality rate and total mortality rate of surgery, did not show a dramatic change during the study periods. Such as the inpatient mortality rate in 2016, 2017, and 2018 was 0.31%, 0.27%, and 0.28%, respectively, which implied that the inpatient mortality rate remained more or less unchanged before and after the C-DRG reform.

### Limitations

There were several limitations to this study. First, although there were no other relevant changes coinciding with the C-DRG reform in Sanming during the study periods, and all the sensitivity analyses showed the robustness of the results, the before-after study design precludes a direct causal interpretation of the results. Second, data about the quality of care for patients with hip fracture, such as information on readmission, postoperative and 1-year mortality, are unavailable. Therefore, it is not certain whether the C-DRG reform affected the quality of care for patients with hip fracture, although the overall quality indicators of public hospitals in Sanming did not show a dramatic change during the study periods. Consequently, the long-term postoperative mortality and readmission should be closely monitored in the future for patients with hip fracture. Third, although there was a performance measurement system to encourage the implementation of clinical pathways and prevent physicians providing inappropriate treatment under the C-DRG payment system, the limits of administrative datasets preclude judgment about appropriateness of clinical pathways for hip fracture. Thus, further comprehensive evaluation is needed. Truly, this database in Sanming was already the most comprehensive and generalizable database in China currently, because Sanming is the pioneer of China's health reform. But the database systems in China still lack more detailed information, compared to those in Europe and America. At this point, the designs of whole database systems need to be improved in the future in China. Fourth, only Sanming as the first pilot city of C-DRG reform was examined; therefore, the results may not be generalized to other areas. Future more extensive evaluation need to be performed, when C-DRG policy are rolled out in more pilot cities in China; and the adoption of DRG payment system for other countries requires adequate modifications to reflect specific country context. Finally, we only used the first year of data after the C-DRG reform in our analyses, and thus these findings cannot be interpreted as long-term effects of the reform.

## Conclusions

C-DRG reform was associated with a decrease in both the absolute amount of OOP payments and OOP payments as a share of total inpatient expenditure, which suggested that the C-DRG reform helped to provide better financial protection for older patients with hip fracture, at least in the first year of C-DRG implementation. The results of this study can help inform policymakers who are developing or plan to reform their payment system to reduce financial burden for patients in other countries with similar systems.

## Supplementary Information

**Additional file 1: Table S1.** Descriptions of C-DRG reform measures.

**Additional file 2: Table S2.** The GLM analysis results of inpatient expenditures and length of stay: Results with year and hospital fixed effects, before and after accounting for clustering effect within hospitals.

**Additional file 3: Table S3.** The association between insurance types and treatment methods of the study population.

**Additional file 4: Table S4.** Unadjusted length of stay by treatment methods.

**Additional file 5: Table S5.** The overall quality indicators of public hospitals in Sanming City during the study periods.

## Data Availability

The datasets and materials used in this study are not publicly available due to limitations of ethical approval involving the patient data and anonymity but are available from the corresponding author on reasonable request.

## Abbreviations

C-DRG: Chinese diagnosis-related group
CCI: Charlson comorbidity index
DRG: Diagnosis-related groups
FFS: Fee-for-service
GLM: Generalized linear models
ICD-10: International Classification of Diseases, 10th Revision
LMICs: Low- and middle-income countries
LOS: Length of stay
OOP: Out-of-pocket
PSM: Propensity score matching
TCM: Traditional Chinese medicine
UHC: Universal health coverage
URRBMI: Urban and Rural Resident Basic Medical Insurance
UEBMI: Urban Employee Basic Medical Insurance
WHO: World Health Organization
