# Supplementary material for: Associations of Chinese diagnosis-related group systems with inpatient expenditures for older people with hip fracture
Source: BMC Geriatr. 2022 Mar 1;22:169. doi: 10.1186/s12877-022-02865-3 (PMC8887083; doi:10.1186/s12877-022-02865-3)
Supplement: Supplementary file 1 — Additional file 1: Table S1. Descriptions of C-DRG reform measures. [file 12877_2022_2865_MOESM1_ESM.docx]

### Supplementary material

**Additional file 1: Table S1.** Descriptions of C-DRG reform measures

| Scope and coverage |
| --- |
| - Under the C-DRG payment system, there are 788 DRGs in 2018. DRGs are a classification system that groups patients according to age, principal diagnosis, secondary diagnoses, treatment methods, and the presence of complications or comorbidities. - C-DRG payment system was eligible for all the public hospitals (secondary and tertiary hospitals). - C-DRG payment system was eligible for all the hospitalized patients except for psychiatric patients. - C-DRG payment system was eligible for all the social insurance schemes, including urban and rural resident basic medical insurance, and urban employee basic medical insurance. |
| Price and payment |
| - Both the insurance reimbursements and patient out-of-pocket payments are prospectively reimbursed by DRG. For example, patient out-of-pocket payments account for 30% and insurance reimbursements account for 70% of a "bundled" payment for each DRG in secondary hospitals. - Payments for cases of outliers depended on cost, which represented only a small minority of cases. - The price for each DRG was based on cost accounting systems to make sure the accuracy of payment reflecting the true cost to minimize risk selection. - Hospitals can keep any savings but also bear financial risk of any cost overrun for hospital patients. |
| Coding and documentation |
| - Multiple diagnoses are embedded into the electronic medical record system, and physicians choose the diagnoses and procedures from the electronic medical record system for each patient, which sequentially can be converted to ICD codes. The physicians or coders do not need to translate narrative diagnoses and procedures into ICD codes directly. - There is a committee to oversee the completeness and accuracy of medical records in all hospitals. Moreover, code checkers with medical backgrounds are responsible for verifying the accuracy of the assigned code for all discharged patients through the electronic medical record system, notifying the hospital of any errors. - A series of training about standardized clinical documentation and coding were performed before the adoption of C-DRG. - A robust health information system was used to detect the accuracy of documentation and coding. And the detected unintended effects, such as the upcoding behaviors, would be penalized. |

Abbreviations: *C-DRG* Chinese diagnosis-related group
