# Supplementary material for: Associations of Chinese diagnosis-related group systems with inpatient expenditures for older people with hip fracture
Source: BMC Geriatr. 2022 Mar 1;22:169. doi: 10.1186/s12877-022-02865-3 (PMC8887083; doi:10.1186/s12877-022-02865-3)
Supplement: Supplementary file 2 — Additional file 2: Table S2. The GLM analysis results of inpatient expenditures and length of stay: Results with year and hospital fixed effects, before and after accounting for clustering effect within hospitals. [file 12877_2022_2865_MOESM2_ESM.docx]

### Supplementary material

**Additional file 2: Table S2.** The GLM analysis results of inpatient expenditures and length of stay: Results with year and hospital fixed effects, before and after accounting for clustering effect within hospitals

| Variable | Ln (Total  expenditure)  (1) | Ln (OOP payments)  (2) | | Ln (OOP% of total expenditure)  (3) | | Ln (Length of stay)  (4) | | Ln (Total  expenditure)  (5) | Ln (OOP payments)  (6) | | Ln (OOP% of total expenditure)  (7) | | Ln (Length of stay)  (8) |  |
| --- | --- | --- | --- | --- | --- | --- | --- | --- | --- | --- | --- | --- | --- | --- |
| C-DRG reform (after vs. before^ref^) | −0.011  (0.024) | | −0.174  (0.027)^***^ | −6.471  (0.396)^***^ | −0.110  (0.024)^***^ | | −0.011  (0.039) | | | −0.174  (0.036)^***^ | | −6.471  (1.344)^***^ | −0.110  (0.032)^**^ | |
| Intercept | 7.948  (0.130)^***^ | | 6.967  (0.148)^***^ | 38.537  (2.173)^***^ | 2.000  (0.130)^***^ | | 7.948  (0.114)^***^ | | | 6.967  (0.154)^***^ | | 38.537  (2.772)^***^ | 2.000  (0.123)^***^ | |
| ^a^Covariates | YES | | YES | YES | YES | | YES | | | YES | | YES | YES | |
| Yearly fixed effects | YES | | YES | YES | YES | | YES | | | YES | | YES | YES | |
| Hospitals fixed effects | YES | | YES | YES | YES | | YES | | | YES | | YES | YES | |
| Clustered standard error | NO | | NO | NO | NO | | YES | | | YES | | YES | YES | |
| R-square | 0.722 | | 0.697 | 0.407 | 0.479 | | 0.722 | | | 0.697 | | 0.407 | 0.479 | |

^**^*p* < 0.01, ^***^*p* < 0.001

Robust standard errors are reported in parentheses in columns (1)-(4); standard errors clustered at the hospital level are reported in parentheses in column (5)-(8)

^a^Covariates included age, gender, insurance types, fracture location, treatment methods, Charlson comorbidity index, hospital levels, and hospital types

Abbreviations: *C-DRG* Chinese diagnosis-related group, *GLM* generalized linear models, *ref* reference group, *OOP* out-of-pocket
