# Supplementary material for: Associations of Chinese diagnosis-related group systems with inpatient expenditures for older people with hip fracture
Source: BMC Geriatr. 2022 Mar 1;22:169. doi: 10.1186/s12877-022-02865-3 (PMC8887083; doi:10.1186/s12877-022-02865-3)
Supplement: Supplementary file 3 — Additional file 3: Table S3. The association between insurance types and treatment methods of the study population. [file 12877_2022_2865_MOESM3_ESM.docx]

### Supplementary material

**Additional file 3: Table S3.** The association between insurance types and treatment methods of the study population

|  | Surgical treatment (HR/IF) | Other treatments | χ^2^ | *p* |
| --- | --- | --- | --- | --- |
| Insurance type, n (%) |  |  | 9.565 | 0.002 |
| URRBMI | 2109 (75.3) | 691 (24.7) |  |  |
| UEBMI | 388 (81.9) | 86 (18.1) |  |  |

Abbreviations: *UEBMI* Urban Employee Basic Medical Insurance, *URRBMI* Urban and Rural Resident Basic Medical Insurance, *HR/IF* hip replacement/internal fixation
