# Supplementary material for: Associations of Chinese diagnosis-related group systems with inpatient expenditures for older people with hip fracture
Source: BMC Geriatr. 2022 Mar 1;22:169. doi: 10.1186/s12877-022-02865-3 (PMC8887083; doi:10.1186/s12877-022-02865-3)
Supplement: Supplementary file 4 — Additional file 4: Table S4. Unadjusted length of stay by treatment methods. [file 12877_2022_2865_MOESM4_ESM.docx]

### Supplementary material

**Additional file 4: Table S4.** Unadjusted length of stay by treatment methods

|  | Surgical treatment (HR/IF)  Mean (SD) | Other treatments  Mean (SD) | *t* | *p* |
| --- | --- | --- | --- | --- |
| Length of stay (days) | 20.42 (10.00) | 8.86 (11.03) | 27.435 | <0.001 |

Abbreviations: *HR/IF* hip replacement/internal fixation
