# Supplementary material for: Associations of Chinese diagnosis-related group systems with inpatient expenditures for older people with hip fracture
Source: BMC Geriatr. 2022 Mar 1;22:169. doi: 10.1186/s12877-022-02865-3 (PMC8887083; doi:10.1186/s12877-022-02865-3)
Supplement: Supplementary file 5 — Additional file 5: Table S5. The overall quality indicators of public hospitals in Sanming City during the study periods. [file 12877_2022_2865_MOESM5_ESM.docx]

### Supplementary material

**Additional file 5: Table S5.** The overall quality indicators of public hospitals in Sanming City during the study periods

| Indicators | Year  2016 | Year  2017 | Year  2018 |
| --- | --- | --- | --- |
| Inpatient mortality rate (%) | 0.31 | 0.27 | 0.28 |
| Total mortality rate of surgery (%) | 0.07 | 0.02 | 0.06 |
| Success rate of emergency treatment (%) | 95.30 | 96.05 | 95.61 |
| Rate of pressure ulcer (%) | 0.017 | 0.036 | 0.029 |
| Number of new technology projects (n) | 114 | 145 | 232 |
